# Effects of a Smartphone-Based Approach-Avoidance Intervention on Chocolate Craving and Consumption: Randomized Controlled Trial

**DOI:** 10.2196/12298

**Published:** 2019-11-01

**Authors:** Adrian Meule, Anna Richard, Radomir Dinic, Jens Blechert

**Affiliations:** 1 Schoen Clinic Roseneck Prien am Chiemsee Germany; 2 Department of Psychiatry and Psychotherapy University Hospital LMU Munich Munich Germany; 3 Department of MultiMediaTechnology Salzburg University of Applied Sciences Puch Austria; 4 Department of Psychology University of Salzburg Salzburg Austria

**Keywords:** food, chocolate, craving, smartphone, mobile phone, mhealth, digital health, eating behavior

## Abstract

**Background:**

Repeatedly pushing high-calorie food stimuli away based on joystick movements has been found to reduce approach biases toward these stimuli. Some studies also found that such avoidance training reduced consumption of high-calorie foods.

**Objective:**

This study aimed to test effects of a smartphone-based approach-avoidance intervention on chocolate craving and consumption, to make such interventions suitable for daily use.

**Methods:**

Within a 10-day period, regular chocolate eaters (n=105, 86% female) performed five sessions during which they continuously avoided (ie, swiped upward) chocolate stimuli (experimental group, n=35), performed five sessions during which they approached and avoided chocolate stimuli equally often (placebo control group, n=35), or did not perform any training sessions (inactive control group, n=35). Training effects were measured during laboratory sessions before and after the intervention period and further continuously through daily ecological momentary assessment.

**Results:**

Self-reported chocolate craving and consumption as well as body fat mass significantly decreased from pre- to postmeasurement across all groups. Ecological momentary assessment reports evidenced no differences in chocolate craving and consumption between intervention days and rest days as a function of the group.

**Conclusions:**

A smartphone-based approach-avoidance training did not affect eating-related and anthropometric measures over and above measurement-based changes in this study. Future controlled studies need to examine whether other techniques of modifying food approach tendencies show an add-on benefit over conventional, monitoring-based intervention effects.

**Trial Registration:**

AsPredicted 8203; https://aspredicted.org/pt9df.pdf.

## Introduction

Training individuals to avoid appetitive stimuli has been found to reduce automatic approach tendencies toward these stimuli. For example, repeatedly pushing pictures of alcoholic beverages away on a screen based on joystick movements has been found to reduce approach biases toward alcohol in heavy drinkers [1] and patients with alcohol use disorder [2,3]. Similar results have been obtained using pictures of high-calorie foods in samples of high trait food cravers [4] or individuals with obesity [5-7]. Although effects on actual consumption behaviors is less consistent [8-10], several studies point toward a decrease in craving for and consumption of appetitive substances through approach-avoidance training [11].

While traditional approach-avoidance tasks (AATs) and training are usually performed with joystick movements in front of a computer monitor, methods that make these techniques suitable for daily use are needed. One possibility for this is to implement AATs or training on smartphones. For example, 2 recent studies used a smartphone-based training during which participants were required to swipe pictures away or toward themselves to reduce body dissatisfaction [12] or procrastination [13]. Although these studies reported promising results (ie, changes in behavior because of the approach-avoidance intervention), interpretation was limited by the use of inactive (waitlist) control groups and by combining the training with conventional face-to-face treatment elements.

The aim of this study was, therefore, to evaluate a smartphone-based approach-avoidance training for reducing food craving and consumption in a randomized, fully controlled trial (ie, by comparing active training effects to placebo and no training groups). As chocolate is the most frequently craved food in Western societies [14,15], we restricted our study to chocolate-containing foods, similar to previous studies on approach-avoidance modification [16-18]. Specifically, participants were randomly assigned to 1 of 3 groups: during a 10-day period, they either performed five training sessions during which they continuously avoided pictures of chocolate-containing foods (upward swipes) and approached pictures of neutral objects (downward swipes; *experimental group*), performed 5 training sessions during which they approached and avoided food and neutral stimuli equally often (*placebo control group*), or did not perform an approach-avoidance training (*inactive control group*). All participants completed an AAT and reported their craving for and consumption of chocolate-containing foods before and after the 10-day period. Furthermore, previous studies found short-term effects of approach-avoidance training on food consumption (eg, reduced chocolate muffin consumption in a taste test immediately after an avoidance training session [18]). To capture such short-lived effects, participants reported their craving for and consumption of chocolate-containing foods on each evening during the 10-day period. This allowed us to examine both short-term training effects by comparing chocolate craving and consumption on intervention versus rest days during the 10-day period and long-term training effects by comparing pre- versus posttest values before and after the 10-day period.

We tested the following preregistered hypotheses (Multimedia Appendix 1):

(1) Similar to findings showing that an approach bias modification training decreased approach bias toward high-calorie foods [4], we expected that approach bias toward chocolate-containing foods would decrease from pre- to posttest only in the experimental group but not in the 2 control groups.

(2) Similar to findings showing that self-monitoring of snacking decreases snack food consumption [19], we expected that self-reported chocolate craving and consumption in the past 10 days would decrease from pre- to posttest in all 3 groups, as all participants were confronted with their chocolate consumption behavior during the study. However, because of craving- and consumption-reducing effects of approach-avoidance training found in previous studies [4,18], we expected that these decreases would be larger in the experimental group than in the placebo control group and the inactive control group.

(3) Performing reaction time tasks involving palatable food pictures usually increases food craving from immediately before to immediately after the task [20,21]. Therefore, we expected that performing a chocolate-related AAT would induce chocolate craving, that is, current chocolate craving would be increased immediately after having performed the task compared with before. At pretest, we expected that these chocolate craving increases during the task would be similar in all 3 groups. As previous findings indicate that approach-avoidance training can decrease such food cue–induced craving [4], we expected that task-induced chocolate craving would be attenuated at posttest in the experimental group but not in the inactive control group. As participants in the placebo control group were confronted with the chocolate pictures more often than participants in the inactive control group, we expected that the placebo group would show an attenuation of task-induced chocolate craving at posttest as well because of habituation. Finally, we hypothesized that current hunger would be unaffected by the intervention, that is, would be similar across groups and measurements.

(4) Given that short-term effects on food consumption have been reported in approach bias modification studies (ie, reduced consumption after a training session [18]), we expected that chocolate craving and consumption would be reduced on intervention days compared with rest days in the experimental group, and this difference would be larger than in the placebo control group.

In addition to these preregistered analyses, we also explored changes in body mass index and body fat mass as a function of group, examined whether any effects were moderated by baseline levels of trait chocolate craving and restrained eating, and tested whether groups differed in awareness of the study’s aims.

## Methods

### Participants

A power analysis was conducted with G*Power version 3.1.9.2 [22] for repeated measures analysis of variance with a within-between interaction. This revealed that a sample size of 102 (ie, n=34 participants per group) would be sufficient to detect a small effect (*f*=0.1), given an alpha level of .05, power of .80, 3 groups, 2 measurements, and a correlation of *r*=.80 between repeated measures.

**Figure 1:**
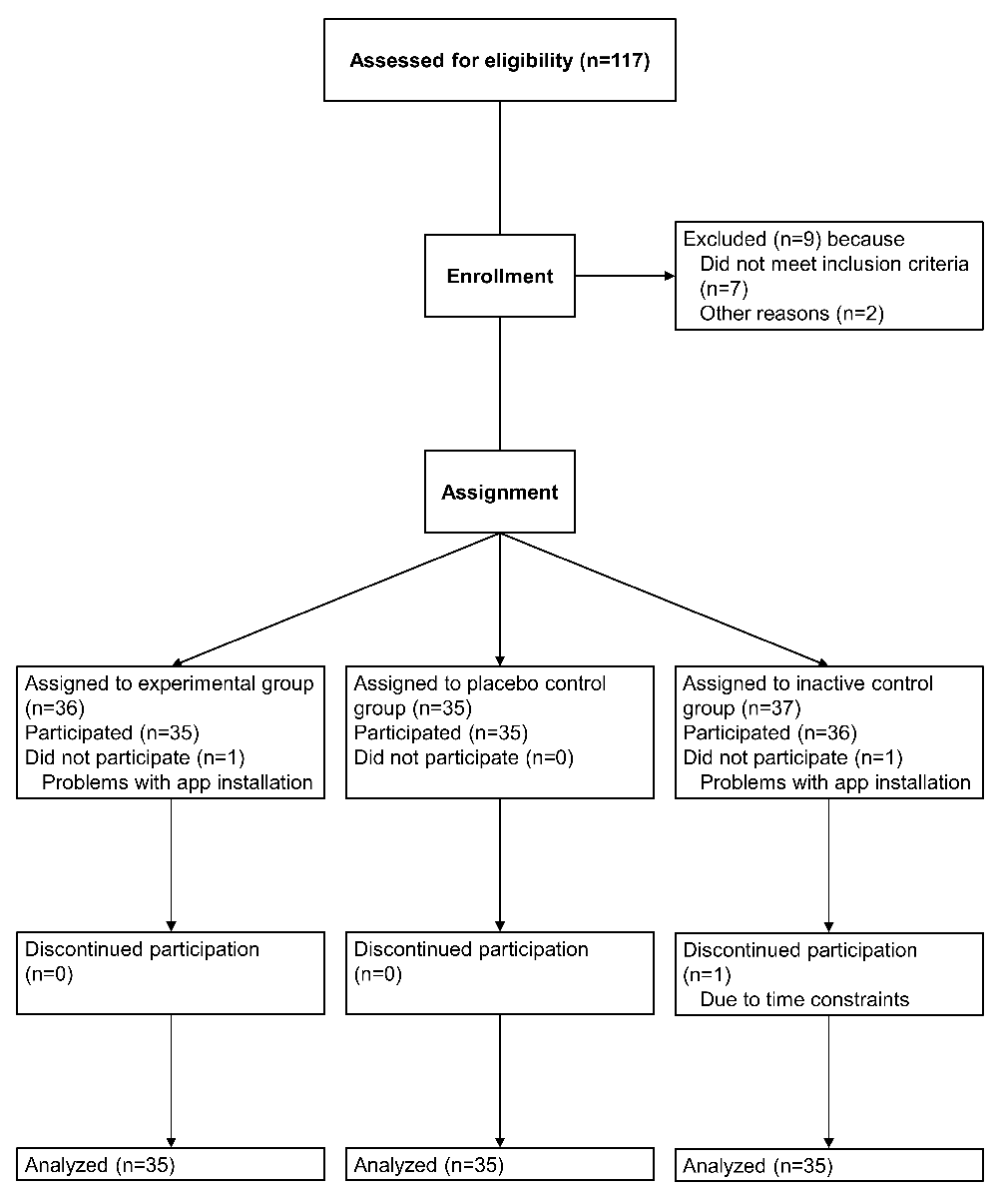
Flow of participants throughout the study. Note that while sample size was n=105 for the majority of analyses, sample size was n=104 for analyses involving body mass index at posttest and n=102 for analyses involving body fat mass at posttest because of missing data.

Participants were recruited at the University of Salzburg and through a local job advertisements website. Inclusion criteria were speaking fluent German, aged between 18 and 50 years, not being pregnant, and not having participated in similar studies in our laboratory. Recruitment advertisements also indicated that participants should be regular chocolate eaters (ie, several times per week) and should not be underweight or currently dieting. A total of 117 individuals responded to the advertisements. A total of 9 participants were excluded before enrollment: 7 participants did not meet inclusion criteria (current pregnancy: n=1, non–German-speaking: n=2, already participated in similar studies in our laboratory: n=4), and 2 participants indicated that they recently decided to refrain from eating chocolate because of lactose intolerance and health reasons (n=2; Figure 1). Of the remaining 108 individuals, 2 did not participate because of technical problems, and 1 discontinued participation (Figure 1). The final sample comprised 105 participants (85.7% female, 90/105) with a mean age of 23.4 years (SD 5.07) and a mean body mass index of 23.3 kg/m² (SD 4.14). The majority of participants had German (52.4%, 55/105) or Austrian (40.0%, 42/105) citizenship and were university students (94.3%, 99/105).

### Materials

#### Approach-Avoidance Task

An AAT was employed to examine whether approach bias toward chocolate-containing foods changed from pre- to posttest as a function of group. The task was programmed in unity (Unity Technologies) and run on a 5-inch SAMSUNG Galaxy J3 smartphone (Samsung Electronics Austria GmbH). A total of 16 pictures of chocolate-containing foods and 16 pictures of nonedible objects were taken from the food pics database [23] (Multimedia Appendix 2). Pictures were matched regarding color, size, brightness, contrast, complexity, recognizability, and familiarity and have previously been used in a joystick-based AAT with which an approach bias toward food was found [24]. The task consisted of 2 blocks: participants were instructed to swipe pictures of food upward (=“away from yourself”) and swipe pictures of objects downward (=“toward yourself”) with the thumb of their dominant hand in 1 block and vice versa in the other block (block order was counterbalanced across participants). Within each block, each picture was presented twice in randomized order. Thus, participants pulled food, pushed food, pulled objects, and pushed objects in 32 trials each, totaling 128 trials. In each trial, 1 picture appeared in the center of the smartphone screen. Similar to joystick-based AATs [25], a zoom effect was employed: picture size increased when the picture was swiped downwards and decreased when the picture was swiped upwards. The picture then disappeared when reaching the border of the screen, and the next trial started (Multimedia Appendix 3).

#### Sociodemographic and Anthropometric Data

Participants indicated their age, sex, handedness, education, and nationality. Body height (in cm) was measured with a wall-mounted stadiometer. Body weight (in kg) and fat mass (in %) were measured with the OMRON Body Composition Monitor BF511 (OMRON Healthcare Europe BV).

#### Chocolate Consumption

To examine whether chocolate consumption changed from pre- to posttest as a function of group, participants responded to the question “How often did you consume chocolate-containing foods in the past ten days?” Responses were recorded on a rating slider anchored 0=*not at all* and 100=*very often*.

#### Food Cravings Questionnaire-Trait-Reduced

The German, chocolate-adapted version of the Food Cravings Questionnaire-Trait-reduced (FCQ-T-r) [26] was used to examine whether groups differed at pretest, whether pretest scores moderated any intervention effects, and whether scores changed from pre- to posttest as a function of group. Participants are usually instructed to indicate how frequently each statement is true for them in general. However, to fit the purpose of this study, participants were instructed to indicate how frequently each statement was true for them in the past 10 days. The scale has 15 items (eg, “If I am craving chocolate, thoughts of eating it consume me” and “It is hard for me to resist the temptation to eat chocolate that is in my reach”), which are scored from 1=*never* to 6=*always*. Internal reliability was α=.894 at pretest and α=.921 at posttest.

#### Food Cravings Questionnaire-State

The German, chocolate-adapted version of the Food Cravings Questionnaire-State (FCQ-S) [26] was used to measure current chocolate craving and hunger before and after the AAT. The scale has 15 items (12 items for the chocolate craving subscale and 3 items for the hunger subscale), which are scored from 1=*strongly disagree* to 5=*strongly agree*. Internal reliabilities of the chocolate craving subscale ranged between α=.873 and α=.930, and internal reliabilities of the hunger subscale ranged between α=.835 and α=.917 in this study.

#### Restraint Scale

The German version of the Restraint Scale [27] was used to examine whether groups differed in dietary restraint and whether dietary restraint moderated any intervention effects. The scale has 10 items, which are scored from 0 to 4 (items 1-4 and 10) and 0 to 3 (items 5-9) with different response options. Internal reliability was α=.715 in this study.

#### Dutch Eating Behavior Questionnaire

The German version of the Dutch Behavior Questionnaire’s (DEBQ’s) restrained eating subscale [28] was used to examine whether groups differed in dietary restraint and whether dietary restraint moderated any intervention effects. The scale has 10 items, which are scored from 1=*never* to 5=*very often*. Internal reliability was α=.879 in this study.

#### Eating Disorder Examination-Questionnaire 8

The German version of the Eating Disorder Examination-Questionnaire 8 (EDE-Q8) [29] was used to examine whether groups differed in eating disorder symptomatology. The scale has 8 items that are scored from 0=*no days*/*never*/*not at all* to 6=*every day*/*every time*/*very much*. Internal reliability was α=.883 in this study.

#### End-of-Day Questions

On each evening during the 10-day period between pre- and posttest, participants answered questions on their smartphone using the application PsyDiary (MultimediaTechnology). Chocolate craving intensity was assessed with the question “How strong was your desire for chocolate-containing foods today (on average)?” Answers were recorded on a rating slider anchored 0=*very weak* and 100=*very strong*. Chocolate craving frequency was assessed with the question “How often did you have a desire for chocolate-containing foods today?” Answers were recorded on a rating slider anchored 0=*not at all* and 100=*very often*. Chocolate consumption quantity was assessed with the question “How many chocolate-containing foods did you consume today?” Answers were recorded on a rating slider anchored 0=*none* and 100=*a great many*. Chocolate consumption frequency was assessed with the question “How often did you consume chocolate-containing foods today?” Answers were recorded on a rating slider anchored 0=*not at all* and 100=*very often*.

#### Debriefing Questions

Awareness of the study’s aims was assessed with the questions “Do you think that the aim of this study was to assess your behavior in relation to chocolate?” and “Do you think that the aim of this study was to change your behavior in relation to chocolate?” Response options for both questions were *yes*, *no*, and *I don’t know*.

### Procedure

The study was approved by the ethical review board of the University of Salzburg, and study design and hypotheses were preregistered at aspredicted.org. The study was advertised as a study on “automatic reactions to chocolate-containing foods in daily life.” That is, participants were not informed that the aim of the study was to change chocolate craving and consumption. Participants were randomly assigned to 1 of the 3 groups and were tested in the laboratory individually.

#### Pretest

At pretest, participants signed informed consent and completed the FCQ-T-r, the question on chocolate consumption in the past 10 days, and the FCQ-S. Afterward, participants practiced the swipe movements in 2 blocks with 10 trials each (which included pictures of animals and household items that were not used in the main task) and then completed the AAT. Then, they completed the FCQ-S again, responded to the sociodemographic questions, and completed the Restraint Scale, the DEBQ, and the EDE-Q8. Subsequently, body height, weight, and fat mass were measured. Finally, participants installed the apps, and the experimenter explained their use, the remaining study procedures, and discussed any open questions. At the end of the day of the pretest, participants received the first prompt (ie, end-of-day questions) to familiarize them with the app (these data were discarded from analyses).

#### Intervention Period

During the 10-day period between the pre- and posttest, all participants received the end-of-day questions on each evening at 9 pm and could respond to the questions until 10 pm. The experimental group additionally performed 5 training sessions (1 session on 5 days each). Training sessions were similar to the AAT used at pre- and posttest, except that pictures of food were always swiped upwards and pictures of objects were always swiped downwards (ie, there was no reversal of instructions between blocks). The placebo control group also performed 5 training sessions (1 session on 5 days each). Here, training sessions were equal to the AAT used at pre- and posttest, that is, pictures of food and objects were swiped upward or downward equally often. In both the experimental and placebo control group, intervention and rest days were pseudorandomized with a maximum of 3 consecutive intervention or rest days. On intervention days, the training session was available between 12 noon and 8 pm. (reminders were sent every 2 hours). The inactive control group did not perform any training sessions.

#### Posttest

At posttest, participants again completed the FCQ-T-r, the question on chocolate consumption in the past 10 days, and the FCQ-S; performed the AAT; and then completed the FCQ-S again in the laboratory. Finally, they completed the debriefing questions, and body weight and fat mass were measured. Participation was reimbursed with course credits or €40. The amount of course credits or money was reduced when participants did not complete all signals (ie, training sessions or end-of-day questions).

### Data Analyses

#### Randomization Check and Compliance

We compared groups regarding baseline characteristics with analyses of variance (age, body mass index, body fat mass, chocolate consumption, FCQ-T-r scores, Restraint Scale scores, DEBQ scores, and EDE-Q8 scores) and Fisher exact tests (sex, handedness, education, and nationality). Furthermore, we compared groups regarding the number of completed training sessions (in %) and completed end-of-day questions (in %) with Kruskal-Wallis tests.

#### Hypothesis 1

Erroneous trials (eg, swipes in the wrong direction) were excluded from analyses. These accounted for 7.27% of all trials at pretest and 10.4% of all trials at posttest. The number of valid trials did not differ between groups (Kruskal-Wallis tests: pretest *P*=.25, posttest *P*=.23). Owing to the task setup, we were able to differentiate between 2 different reaction times: the time between picture appearance and participants’ first touch on the screen (*touching time*) and the time between participants’ first touch on the screen and picture disappearance (*dragging time*). Bootstrapped split-half reliability estimates for each condition (pull food, push food, pull objects, and push objects) were obtained using the R package *splithalf* [30] performing 5000 random splits. Reliability estimates for touching time ranged between *r*=.70 and .77 (Spearman-Brown-corrected *r*_sb_=.82-.87) at pretest and between *r*=.79 and .81 (Spearman-Brown-corrected *r*_sb_=.88-.90) at posttest. Reliability estimates for dragging time ranged between *r*=.69 and .82 (Spearman-Brown-corrected *r*_sb_=.82-.90) at pretest and between *r*=.63 and .83 (Spearman-Brown-corrected *r*_sb_=.77-.90) at posttest.

In line with joystick-based AAT studies [25], median reaction times were calculated. As outlined in the preregistration, 3×2×2×2 analyses of variance for repeated measures were calculated with median reaction time data as dependent variables, *group* (experimental vs placebo control vs inactive control) as between-subjects factor and *measurement* (pre- vs posttest), *stimulus* (food vs objects), and *direction* (pull vs push) as within-subjects factors. This was done separately for touching time and for dragging time (which was not explicitly specified in the preregistration).

#### Hypothesis 2

As outlined in the preregistration, 3×2 analyses of variance for repeated measures were calculated with self-reported chocolate consumption and FCQ-T-r scores as dependent variables, *group* (experimental vs placebo control vs inactive control) as between-subjects factor, and *measurement* (pre- vs posttest) as within-subjects factor.

#### Hypothesis 3

As outlined in the preregistration, 3×2×2 analyses of variance for repeated measures were calculated with FCQ-S scores (current chocolate craving and hunger) as dependent variables, *group* (experimental vs placebo control vs inactive control) as between-subjects factor, and *measurement* (pre- vs posttest) and *task* (before vs after the task) as within-subjects factors.

#### Hypothesis 4

Responses to the end-of-day questions on intervention days on which participants did not complete the training session were excluded from analyses. These accounted for 47 signals (6.71%) of the possible 700 signals (10 days×70 participants [experimental + placebo control group]). As outlined in the preregistration, we applied linear mixed models using the R package *lme4* [31] to analyze the nested, longitudinal structure of the data. *Days* (0=rest day, 1=intervention day; Level 1) and *group* (0=experimental group, 1=placebo control group; Level 2) and their cross-level interaction *group* × *days* were used as predictors for chocolate craving intensity/frequency and for chocolate consumption quantity/frequency. We further explored whether pretest scores of the FCQ-T-r*,* Restraint Scale, and DEBQ at level 2 would modulate any effects. The level 1 predictor *days* was entered uncentered to the models, and the level 2 predictors *group*, *FCQ-T-r*, *Restraint Scale*, and *DEBQ* were grand-mean centered. The intercepts of all models were allowed to vary randomly. The data files and R-script for these analyses can be found in Multimedia Appendix 4.

#### Exploratory Analyses

Analyses of variance for repeated measures with *group* (experimental vs placebo control vs inactive control) as between-subjects factor and *measurement* (pre- vs posttest) as within-subjects factor were calculated to examine changes in body mass index and body fat mass as a function of group. Moderation analyses were calculated with PROCESS [32] to examine whether FCQ-T-r scores at pretest, Restraint Scale scores, and DEBQ scores moderated any effects of group on chocolate consumption, body mass index, and body fat mass at posttest while controlling for pretest values. Restraint Scale scores and DEBQ scores were also tested as moderators of effects of group on FCQ-T-r scores at posttest while controlling for FCQ-T-r scores at pretest. Fisher exact tests were calculated to compare groups regarding the 2 debriefing questions. These analyses were not included in the preregistration protocol.

## Results

### Randomization Check and Compliance

Groups did not differ in any baseline characteristics (Table 1). Compliance was high for both completion of the training sessions (86.6%) and completion of the end-of-day questions (85.8%) and did not differ between groups (Table 1).

**Table 1:** Means and frequencies of study variables at pretest and compliance rates during the intervention phase as a function of the group (N=105).

Study variables	Experimental group (n=35)	Placebo control group (n=35)	Inactive control group (n=35)	Test statistics	*P* value
Age (years), mean (SD)	22.7 (3.36)	24.1 (6.13)	23.5 (5.37)	*F*_2,102_=0.64, η_p_²=.012	.53
Sex (female), n (%)	30 (85.7)	32 (91.4)	28 (80.0)	χ^2^=1.8, Φ=.133	.45
Handedness (right-handed), n (%)	28 (80.0)	32 (91.4)	33 (94.3)	χ^2^=3.5, Φ=.194	.23
Education (students), n (%)	33 (94.3)	32 (91.4)	34 (97.1)	χ^2^=1.1, Φ=.101	.87
Nationality (German), n (%)	16 (45.7)	21 (60.0)	18 (51.4)	χ^2^=3.5, Φ=.184	.47
Body mass index (kg/m²), mean (SD)	23.5 (4.90)	23.3 (3.62)	23.0 (3.89)	*F*_2,102_=0.16, η_p_²=.003	.86
Body fat mass (%), mean (SD)	31.6 (9.91)	32.8 (7.52)	29.5 (9.00)	*F*_2,102_=1.29, η_p_²=.025	.28
Chocolate consumption (self-report), mean (SD)	55.6 (20.9)	61.5 (21.3)	58.1 (22.8)	*F*_2,102_=0.65, η_p_²=.013	.52
Food Cravings Questionnaire-Trait-reduced (chocolate version), mean (SD)	41.4 (8.54)	41.2 (10.5)	44.2 (12.2)	*F*_2,102_=0.90, η_p_²=.017	.41
Restraint Scale, mean (SD)	11.5 (5.05)	12.0 (4.53)	11.7 (4.69)	*F*_2,102_=0.11, η_p_²=.002	.89
Dutch Eating Behavior Questionnaire (restrained eating subscale), mean (SD)	2.04 (0.80)	2.03 (0.60)	2.16 (0.65)	*F*_2,102_=0.38, η_p_²=.007	.68
Eating Disorder Examination-Questionnaire 8, mean (SD)	0.97 (1.11)	1.00 (0.72)	1.23 (1.08)	*F*_2,102_=0.75, η_p_²=.014	.48
Training sessions compliance (%), mean (SD)	89.1 (17.7)	84.0 (19.3)	—^a^	—	.22
End-of-day questions compliance (%), mean (SD)	88.0 (17.5)	88.6 (16.1)	80.6 (25.6)	—	.22

^a^Not applicable.

### Hypothesis 1

#### Touching Time

A main effect of *direction* (*F*_1,102_=13.3; *P*<.001; η_p_²=.115) indicated that participants touched the target stimuli faster in pull trials (mean 599 ms, SD 55.3) than in push trials (mean 606 ms, SD 56.0). There were significant main effects of *measurement* and *stimulus* and interaction effects *measurement* × *stimulus* and *group* × *measurement* (all *P*s<.001), which were qualified by a significant interaction *group* × *measurement* × *stimulus* (*F*_2,102_=4.82; *P*=.01; η_p_²=.086). However, as this interaction effect was small, did not include any direction effects, and post-hoc comparisons were inconclusive, it was not further interpreted. More information and a graphical depiction can be found in the supplementary material (Figure S1 in Multimedia Appendix 5). There was no significant main effect of *group* (*F*_2,102_=2.17; *P*=.12; η_p_²=.041) and no other significant interaction effects (all *P*s>.16).

#### Dragging Time

A main effect of *stimulus* (*F*_1,102_=9.46; *P*=.003; η_p_²=.085) indicated that participants swiped food pictures (mean 248 ms, SD 44.5) faster than object pictures (mean 252 ms, SD 53.3). There were no other significant main or interaction effects (all *P* values>.05).

### Hypothesis 2

#### Chocolate Craving

A main effect of *measurement* (*F*_1,102_=11.7; *P*=.001; η_p_²=.103) indicated that FCQ-T-r scores decreased from pretest (mean 42.3, SD 10.5) to posttest (mean 40.1, SD 11.7). There was no significant main effect of *group* (*F*_2,102_=0.48; *P*=.62; η_p_²=.009) and no significant interaction *group* × *measurement* (*F*_2,102_=0.79; *P*=.46; η_p_²=.015).

#### Chocolate Consumption

A main effect of *measurement* (*F*_1,102_=10.3; *P*=.002; η_p_²=.092) indicated that self-reported chocolate consumption decreased from pretest (mean 58.4, SD 21.6) to posttest (mean 51.8, SD 20.2). There was no significant main effect of *group* (*F*_2,102_=0.35; *P*=.71; η_p_²=.007) and no significant interaction *group* × *measurement* (*F*_2,102_=0.84; *P*=.44; η_p_²=.016).

### Hypothesis 3

#### Current Chocolate Craving

A main effect of *task* (*F*_1,102_=20.7; *P*<.001; η_p_²=.169) indicated that FCQ-S craving scores increased from before (mean 28.0, SD 7.55) to after the task (mean 29.5, SD 8.65). A main effect of *measurement* (*F*_1,102_=17.6; *P*<.001; η_p_²=.147) indicated the FCQ-S craving scores decreased from pretest (mean 30.1, SD 8.01) to posttest (mean 27.4, SD 9.20). There was no significant main effect of *group* (*F*_2,102_=1.06; *P*=.35; η_p_²=.020) and no significant interaction effects (all *P* values>.46).

#### Hunger

A main effect of *task* (*F*_1,102_=11.0; *P*=.001; η_p_²=.098) indicated that FCQ-S hunger scores increased from before (mean 7.91, SD 2.74) to after the task (mean 8.20, SD 3.01). There was no significant main effect of *measurement* (*F*_1,102_=1.36; *P*=.25; η_p_²=.013), no significant main effect of *group* (*F*_2,102_=2.46; *P*=.09; η_p_²=.046), and no significant interaction effects (all *P* values>.43).

### Hypothesis 4

#### Chocolate Craving Intensity and Frequency

There was no significant effect of intervention versus rest days as a function of group (see Table S1 in Multimedia Appendix 5). Higher FCQ-T-r scores at pretest related to higher chocolate craving intensity and frequency, independent of days and group (Table S2). Restrained eating did not relate to chocolate craving intensity or frequency and did not interact with days or group (Table S3, Table S4).

#### Chocolate Consumption Quantity and Frequency

There was no significant effect of intervention versus rest days as a function of group (Table S5). Higher FCQ-T-r scores at pretest related to higher chocolate consumption quantity and frequency, independent of days and group (Table S6). In addition, a significant *days* × *FCQ-T-r* interaction indicated that participants with high trait chocolate craving scores consumed chocolate-containing foods more frequently on intervention than on rest days, irrespective of group (Table S6). Restrained eating did not relate to chocolate craving quantity or frequency and did not interact with days or group (Table S7, Table S8).

### Exploratory Analyses

#### Body Mass Index

There were no significant main effects and no interaction effect *group* × *measurement* (all *P* values>.56).

#### Body Fat Mass

A main effect of *measurement* (*F*_1,99_=4.43; *P*=.04; η_p_²=.043) indicated that body fat mass decreased from pretest (mean 31.4, SD 8.49) to posttest (mean 31.1, SD 8.73). There was no significant main effect of *group* (*F*_2,99_=0.80; *P*=.45; η_p_²=.016) and no significant interaction *group* × *measurement* (*F*_2,99_=0.30; *P*=.74; η_p_²=.006).

#### Moderation Analyses

There were no significant interaction effects between *group* and *FCQ-T-r*, *Restraint Scale*, and *DEBQ* scores at pretest (all *P* values>.24).

#### Debriefing Questions

A total of 93 participants (88.6%, 93/105) indicated that they thought the aim of the study was to assess their behavior in relation to chocolate, 4 participants (3.8%, 4/105) did not think so, and 8 participants (7.6%, 8/105) indicated that they did not know. There were no significant differences between groups (χ^2^=4.6; *P*=.30; Φ=.224). A total of 29 participants (27.6%, 29/105) indicated that they thought the aim of the study was to change their behavior in relation to chocolate, 61 participants (58.1%, 61/105) did not think so, and 15 participants (14.3%, 15/105) indicated that they did not know. Here, responses did significantly differ between groups (χ^2^=9.63; *P*=.04; Φ=.317): more participants in the inactive control group (n=26) did not think that the study’s aim was to change their behavior than participants in both the experimental group (n=18) and the placebo control group (n=17), whereas the latter 2 groups did not differ from each other (based on follow-up *z* tests using α=.05).

## Discussion

### Summary of Results

This study examined effects of a smartphone-based approach-avoidance intervention on approach bias toward chocolate-containing foods and chocolate craving/consumption relative to placebo and no training conditions. The 3 groups were well matched at baseline, treatment adherence was high (87% completed training sessions), and study attrition was low. All dependent measures evidenced good-to-excellent reliability. However, a smartphone-based AAT did neither reveal an approach bias toward chocolate-containing foods at baseline nor a modulation through training. In fact, chocolate craving and consumption decreased throughout the study period in all 3 groups. This self-report finding was corroborated in that participants in all groups lost body fat. Crucially, only a minority of participants thought that this study’s aim was to change their behavior, suggesting that these effects were not because of demand characteristics. Comparing chocolate craving and consumption on intervention versus rest days did not reveal any short-term effects of the training.

### Measuring and Modifying Approach-Avoidance Tendencies With Swipe Movements

To the best of our knowledge, this is the first study that aimed at measuring and changing an approach bias toward food stimuli based on swipe movements on smartphones. Although there are similar studies that examined effects of smartphone-based approach-avoidance training with swipe movements on procrastination and body dissatisfaction [12,13], these studies did not measure effects of the training on approach-avoidance tendencies. Thus, the lack of finding and modifying an approach bias toward chocolate-containing foods may be related to an insensitivity of our newly developed task to detect such effects. However, several arguments speak against such an interpretation. First, we used the same stimuli with which an approach bias toward food was detected in a comparable sample with a joystick-based AAT [24]. Second, the AAT in this study had moderate-to-good internal reliability [33] and, thus, unreliability of the task is unlikely to account for the current lack of findings. Third, increasing evidence indicates that the type of arm movements (flexion and extension; [34]) or distance change [35] is not essential for measuring or modifying approach-avoidance inclinations. For example, it has been found that upward and downward movements or framing actions as approach and avoidance suffice to modify stimulus evaluations [36]. Nevertheless, future research needs to determine whether other techniques such as moving the smartphone toward and away with arm movements [37,38] or using tilt movements [39] are better suited for detecting and changing approach-avoidance tendencies with smartphones. In addition, it has recently been found that combining approach-avoidance actions with affective feedback produced stronger changes in food choices than conventional approach-avoidance training [40]. Thus, using such consequence-based approach-avoidance training may similarly enhance training effects with smartphone-based implementations.

### Effects of Monitoring Food Intake

Another consideration is that—even if the approach-avoidance training had an effect—it may have been masked by the general decreases in outcome variables across the study period that were observed regardless of group assignment. Specifically, we included daily end-of-day-questions in the study design to be able to examine short-term effects (ie, on the same day) of the single training sessions. However, these questions may have acted as a type of ecological momentary intervention [41]. For example, it has been shown that keeping a daily snack diary reduced snacking frequency, suggesting that cue monitoring suffices to decrease unhealthy food intake (irrespective of additional intervention modules; [42]), potentially through increased awareness for one’s eating behavior. In fact, it has been found that self-monitoring in terms of completing a record of snacking once per day in the evening decreased snack food consumption even in samples that are not particularly motivated to change their behavior [19]. Thus, we cannot fully exclude the possibility that the intervention may have effects—albeit small—on eating behavior that were masked by effects of monitoring food intake.

### Limitations

Interpretation of results needs to consider the sample investigated in this study. Although we included both men and women with a body mass index ranging from underweight to obese, the majority of the sample were normal-weight women. It has been previously suggested that successful retraining of appetitive reactions and consumption behaviors may primarily be found in clinical samples [9]. Although we investigated a nonclinical sample, it is worth noting that our participants had above-average mean scores (>40; Table 1) on the FCQ-T-r (mean scores were 35 in study 1 and 34 in study 2 in the validation studies; [26]), and their eating behavior was clearly impacted throughout the study period (ie, measures were sensitive to detect training-induced changes). This renders insufficient levels of trait chocolate craving as an explanation for these findings unlikely.

Several other methodological considerations might account for these results. For example, although we selected food stimuli with which we have previously detected an approach bias in a comparable sample using a joystick-based task [24], it may be that approach-avoidance training work better when using personalized stimuli, that is, pictures of foods that participants actually crave and consume regularly in their daily life. In related research on attentional bias, for example, it has been found that internal reliability of reaction time tasks can be increased when personalized stimuli are used [43]. Furthermore, we used relatively few training sessions (5), which may have been insufficient to produce meaningful changes in approach bias and eating behavior. However, evidence from joystick-based approach-avoidance training suggest that few sessions suffice to detect such effects in relation to alcohol [44]. Yet, other smartphone-based studies did indeed use more frequent training sessions [12,13]. Thus, the number of training sessions required in smartphone-based approach-avoidance training need further examination. Finally, although we instructed participants regarding the meaning of upward and downward swipe movements, we did not assess whether they actually perceived the movements as pushing or pulling the pictures away from or toward themselves. Therefore, we cannot rule out the possibility that participants did not perceive the movements as intended, which could explain the lack of finding an approach bias and training effects.

### Conclusions

Repeatedly avoiding chocolate-containing foods in terms of (zoom out) upward swipe movements on smartphones did not change behavior related to these foods in this study. Owing to several methodological considerations, there is an urgent need for future research that determines the most effective way of measuring and changing approach-avoidance tendencies in daily life. General decreases in chocolate craving and consumption as well as body fat mass in this study may be because of the generally raised awareness of chocolate consumption throughout the study period. Thus, receiving daily prompts for monitoring food intake may be a cheap and efficient way to normalize food intake in individuals with eating disorders and facilitate weight loss in individuals with obesity.

## Appendix

Multimedia Appendix 1 Preregistration.

Multimedia Appendix 2 Stimuli.

Multimedia Appendix 3 Example trials video.

Multimedia Appendix 4 Study data.

Multimedia Appendix 5 Supplementary material.

Multimedia Appendix 6 CONSORT-EHEALTH checklist (V 1.6.1).

## Abbreviations

AAT: approach-avoidance task
DEBQ: Dutch Behavior Questionnaire
EDE-Q8: Eating Disorder Examination-Questionnaire 8
EMA: ecological momentary assessment
FCQ-S: Food Cravings Questionnaire-State
FCQ-T-r: Food Cravings Questionnaire-Trait-reduced
