# Supplementary material for: Effects of a Smartphone-Based Approach-Avoidance Intervention on Chocolate Craving and Consumption: Randomized Controlled Trial
Source: JMIR Mhealth Uhealth. 2019 Nov 1;7(11):e12298. doi: 10.2196/12298 (PMC6858611; doi:10.2196/12298)
Supplement: Multimedia Appendix 1 [file mhealth_v7i11e12298_app1.pdf]

AsPredicted
Pre-Registration made easy

A food-related, smartphone-based approach–avoidance intervention (#8203)

Author(s)
Adrian Meule (University of Salzburg) - adrian.meule@sbg.ac.at
Anna Richard (University of Salzburg) - anna.richard@sbg.ac.at
Radomir Dinic (Salzburg University of Applied Sciences) - radomir.dinic@sbg.ac.at
Jens Blechert (University of Salzburg) - jens.blechert@sbg.ac.at

Created: 02/05/2018 02:48 AM (PT)
Public: 08/24/2018 03:44 AM (PT)

1) Have any data been collected for this study already?
No, no data have been collected for this study yet.

2) What's the main question being asked or hypothesis being tested in this study?
Main question:
Does a chocolate-containing food-related, smartphone-based approach–avoidance intervention reduce approach bias towards chocolate-containing food, chocolate craving, and chocolate consumption?

Hypotheses:
1. Approach bias towards chocolate-containing food will decrease from pre- to post-measurement only in the intervention group, but not in the two control groups.
2. As all three groups are confronted with their chocolate consumption behavior during the study, self-reported chocolate craving and consumption in the past ten days will decrease from pre- to post-measurement in all three groups. These decreases will be larger in the intervention group than in the placebo control group and the decreases in the placebo control group will be larger than in the inactive control group.
3. Performing a chocolate-related approach–avoidance task will induce chocolate craving, that is, current chocolate craving will be increased after having performed the task compared to before. This task-induced chocolate craving will be attenuated at post test in the intervention group and in the placebo control group, but not in the inactive control group. At pre test, chocolate craving increases during the task will be similar in all three groups. At post test, chocolate craving increases during the task will be smaller in the intervention group than in the placebo control group and will be smaller in the placebo control group than in the inactive control group. Current hunger will be unaffected by the intervention, that is, will be similar across groups and measurements.
4. In the intervention group and placebo control group, chocolate craving and consumption will be reduced on intervention days compared to rest days. This difference between intervention and rest days will be larger in the intervention group than in the placebo control group.

3) Describe the key dependent variable(s) specifying how they will be measured.
Approach–avoidance bias will be measured with a reaction time-based approach–avoidance task on a smartphone, during which pictures of chocolate-containing foods and non-edible objects are swiped upwards and downwards equally often.
Chocolate craving in the past ten days will be measured with the chocolate version of the Food Cravings Questionnaire-Trait-reduced. Chocolate consumption in the past ten days will be measured with a single question and rated on a scale from 0–100.
Current chocolate craving and hunger will be measured before and after performing the approach–avoidance task with the chocolate version of the Food Cravings Questionnaire-State.
Chocolate craving and consumption in daily life will be measured with single questions rated on a scale from 0–100 in the evening of each day during a ten day period.

4) How many and which conditions will participants be assigned to?
Participants will be assigned to one of three groups (i.e., there are three between-subjects conditions). This assignment will be randomized with www.randomizer.org.
In the intervention group, participants will complete five sessions of an approach–avoidance training on their smartphone on five days (i.e., one training session per day) within a ten day period. Which five days of the ten days will be intervention days and which five days will be rest days will be pseudorandomized (not more than three intervention/rest days in a row). During a training session, pictures of chocolate-containing foods are swiped upwards while pictures of non-edible objects are swiped downwards. Furthermore, participants will complete questions in the evening about their chocolate craving and consumption on each day for 10 days.
In the placebo control group, participants will complete five sessions of a placebo approach–avoidance training on their smartphone on five days (i.e., one training session per day) within a ten day period. Which five days of the ten days will be intervention days and which five days will be rest days will be pseudorandomized (not more than three intervention/rest days in a row). During a placebo training session, pictures of chocolate-containing foods and non-edible objects are swiped upwards and downwards equally often. Furthermore, participants will complete questions in the evening about their chocolate craving and consumption on each day for 10 days.
In the inactive control group, participants will only complete questions in the evening about their chocolate craving and consumption on each day for 10 days without any approach–avoidance training.
Before and after the 10 day period, all participants will be tested individually in the laboratory during a pre- and posttest, during which they perform an approach–avoidance task, report their chocolate craving and consumption during the past ten days and report their current chocolate craving and hunger.

Available at https://aspredicted.org/pt9df.pdf
(Permanently archived at http://web.archive.org/web/*/https://aspredicted.org/pt9df.pdf)

AsPredicted
Pre-Registration made easy

5) Specify exactly which analyses you will conduct to examine the main question/hypothesis.
Approach–Avoidance Task:
A 3 x 2 x 2 x 2 analysis of variance for repeated measures will be calculated with reaction time as dependent variable, group (intervention vs. placebo vs. inactive) as between-subjects factor, and time (pretest vs. posttest), stimulus type (food vs. objects), and direction (upward swipe vs. downward swipe) as within-subjects factors.

Self-reported chocolate craving and consumption:
3 x 2 analyses of variance for repeated measures will be calculated with craving or consumption as dependent variable, group (intervention vs. placebo vs. inactive) as between-subjects factor, and time (pretest vs. posttest) as within-subjects factor.

Current chocolate craving and hunger:
3 x 2 x 2 analyses of variance for repeated measures will be calculated with current chocolate craving or hunger as dependent variable, group (intervention vs. placebo vs. inactive) as between-subjects factor, and time (pretest vs. posttest) and task (before vs. after the task) as within-subjects factors.

Ecological momentary assessment questions:
Multilevel modelling will be used for comparing chocolate craving and consumption on intervention days vs. rest days in the intervention group and placebo control group (as the inactive control group does not have any intervention days). Craving or consumption will be used as dependent variable, days (intervention vs. rest) on level 1, and group (intervention vs. placebo vs. inactive) on level 2. On an exploratory basis, the influence of baseline scores of chocolate craving and restrained eating will also be examined at level 2.

6) Describe exactly how outliers will be defined and handled, and your precise rule(s) for excluding observations.
For the approach–avoidance task, data will be excluded when incorrect trials constitute more than 35% of all trials (cf. Wiers et al., 2011).
For the questionnaire data, outliers will be examined with visual inspection of box plots and scatterplots and with this online tool: http://contchart.com/outliers.aspx. Single outliers will be excluded when they clearly drive an effect that is vanished when that outlier is excluded. When excluding an outlier does not change interpretion of results, it will not be excluded.

7) How many observations will be collected or what will determine sample size? No need to justify decision, but be precise about exactly how the number will be determined.
For each of the three groups, 30-35 participants will be tested. According to a power analysis, this sample size will enable detecting small effects around d = 0.3–0.4.

8) Anything else you would like to pre-register? (e.g., secondary analyses, variables collected for exploratory purposes, unusual analyses planned?)

Available at https://aspredicted.org/pt9df.pdf
(Permanently archived at http://web.archive.org/web/*/https://aspredicted.org/pt9df.pdf)
