# Supplementary material for: Effects of a Smartphone-Based Approach-Avoidance Intervention on Chocolate Craving and Consumption: Randomized Controlled Trial
Source: JMIR Mhealth Uhealth. 2019 Nov 1;7(11):e12298. doi: 10.2196/12298 (PMC6858611; doi:10.2196/12298)
Supplement: Multimedia Appendix 5 [file mhealth_v7i11e12298_app5.pdf]

Supplementary material

Table S1

Standardized estimates (B), confidence intervals (CI), standard errors (SE), and p-values for the linear mixed models with the predictors days (0 = rest day, 1 = intervention day), group (0 = experimental group, 1 = placebo control group), and their interaction to predict chocolate craving intensity and frequency

	Chocolate craving intensity				Chocolate craving frequency			
	B	*CI*	*SE*	*p*	*B*	*CI*	*SE*	*p*
**Fixed Parts**								
(Intercept)	32.1	27.1 – 37.2	2.59	<.001	25.9	20.9 – 30.9	2.55	<.001
Days	1.41	−2.83 – 5.65	2.16	.517	3.49	−0.46 – 7.44	2.01	.087
Group	0.11	−7.04 – 7.25	3.65	.976	2.87	−4.17 – 9.90	3.59	.427
Days × Group	−0.98	−7.03 – 5.08	3.09	.753	−2.10	−7.75 – 3.54	2.88	.467
**Random Parts**								
σ^2	339				294			
$\tau_{00,\ participants}$	154				157			
$N_{participants}$	70				70			
Observations	580				580			
R^2 / $\Omega_0{}^2$	.385 / .368				.423 / .410			

Table S2

Standardized estimates (B), confidence intervals (CI), standard errors (SE), and p-values for the linear mixed models with the predictors days (0 = rest day, 1 = intervention day), group (0 = experimental group, 1 = placebo control group), pretest scores of the Food Cravings Questionnaire–Trait–reduced (chocolate version), and their interactions to predict chocolate craving intensity and frequency

	Chocolate craving intensity				Chocolate craving frequency			
	B	*CI*	*SE*	*p*	*B*	*CI*	*SE*	*p*
**Fixed Parts**								
(Intercept)	33.2	29.1 – 37.3	2.10	<.001	26.7	22.5 – 30.9	2.13	<.001
Days	1.75	−2.54 – 6.03	2.19	.426	4.04	0.05 – 8.03	2.04	.050
Group	0.12	−5.65 – 5.89	2.94	.968	3.11	−2.76 – 8.99	2.30	.302
Food Cravings Questionnaire–Trait–reduced (chocolate version)	11.4	6.37 – 16.5	2.58	<.001	8.26	3.12 – 13.4	2.63	.002
Days × Group	−1.34	−7.44 – 4.76	3.11	.668	−2.57	−8.25 – 3.11	2.90	.378
Days × Food Cravings Questionnaire–Trait–reduced (chocolate version)	1.17	−4.18 – 6.51	2.73	.670	3.66	−1.32 – 8.63	2.54	.153
Group × Food Cravings Questionnaire–Trait–reduced (chocolate version)	−2.96	−9.45 – 3.54	3.32	.375	0.43	−6.18 – 7.04	3.37	.899
Days × Group × Food Cravings Questionnaire–Trait–reduced (chocolate version)	−0.58	−7.47 – 6.30	3.51	.869	−3.01	−9.41 – 3.40	3.27	.361
**Random Parts**								
σ^2	340				294			
$\tau_{00,\ \text{participants}}$	71.9				87.5			
$N_{\text{participants}}$	70				70			
Observations	580				580			
R^2 / Ω_0^2	.367 / .357				.414 / .405			

Table S3

Standardized estimates (B), confidence intervals (CI), standard errors (SE), and p-values for the linear mixed models with the predictors days (0 = rest day, 1 = intervention day), group (0 = experimental group, 1 = placebo control group), pretest scores of the Restraint Scale, and their interactions to predict chocolate craving intensity and frequency

	Chocolate craving intensity				Chocolate craving frequency			
	B	*CI*	*SE*	*p*	*B*	*CI*	*SE*	*p*
**Fixed Parts**								
(Intercept)	32.1	27.0 – 37.2	2.61	<.001	25.9	20.9 – 30.9	2.56	<.001
Days	1.35	−2.91 – 5.61	2.17	.536	3.49	−0.48 – 7.46	2.02	.089
Group	0.27	−6.92 – 7.46	3.67	.942	3.10	−3.96 – 10.2	3.60	.392
Restraint Scale	−0.60	−5.48 – 4.29	2.49	.812	−0.75	−5.53 – 4.04	2.44	.761
Days × Group	−0.88	−6.96 – 5.20	3.10	.778	−2.11	−7.78 – 3.56	2.89	.468
Days × Restraint Scale	−1.10	−5.18 – 2.99	2.08	.600	−0.17	−3.97 – 3.64	1.94	.933
Group × Restraint Scale	−2.01	−9.31 – 5.29	3.72	.591	−2.86	−10.0 – 4.29	3.65	.435
Days × Group × Restraint Scale	0.88	−5.21 – 6.98	3.11	.777	0.94	−4.75 – 6.62	2.90	.748
**Random Parts**								
σ^2	340				295			
$\tau_{00, \text{participants}}$	156				157			
$N_{\text{participants}}$	70				70			
Observations	580				580			
R^2 / Ω_0^2	.385 / .369				.423 / .410			

Table S4

Standardized estimates (B), confidence intervals (CI), standard errors (SE), and p-values for the linear mixed models with the predictors days (0 = rest day, 1 = intervention day), group (0 = experimental group, 1 = placebo control group), pretest scores of the Dutch Eating Behavior Questionnaire (restrained eating subscale), and their interactions to predict chocolate craving intensity and frequency

	Chocolate craving intensity				Chocolate craving frequency			
	B	*CI*	*SE*	*p*	*B*	*CI*	*SE*	*p*
**Fixed Parts**								
(Intercept)	32.1	27.0 – 37.3	2.63	<.001	25.9	20.9 – 31.0	2.58	<.001
Days	1.41	−2.83 – 5.65	2.16	.516	3.50	−0.46 – 7.45	2.02	.087
Group	0.06	−7.19 – 7.32	3.70	.987	2.80	−4.34 – 9.94	3.64	.444
Dutch Eating Behavior Questionnaire (restrained eating subscale)	0.32	−4.15 – 4.78	2.28	.889	−0.59	−4.98 – 3.81	2.24	.794
Days × Group	−0.90	−6.96 – 5.16	3.09	.773	−2.03	−7.68 – 3.62	2.88	.483
Days × Dutch Eating Behavior Questionnaire (restrained eating subscale)	−0.87	−4.46 – 2.73	1.83	.638	0.60	−2.75 – 3.95	1.71	.728
Group × Dutch Eating Behavior Questionnaire (restrained eating subscale)	−1.20	−8.63 – 6.22	3.79	.752	−1.01	−8.32 – 6.30	3.73	.787
Days × Group × Dutch Eating Behavior Questionnaire (restrained eating subscale)	3.95	−2.18 – 10.1	3.13	.210	2.19	−3.53 – 7.91	2.92	.455
**Random Parts**								
σ^2		339				294		
$\tau_{00,\text{ participants}}$		161				163		
$N_{\text{participants}}$		70				70		
Observations		580				580		
R^2 / Ω_0^2		.388 / .372				.425 / .412		

Table S5

Standardized estimates (B), confidence intervals (CI), standard errors (SE), and p-values for the linear mixed models with the predictors days (0 = rest day, 1 = intervention day), group (0 = experimental group, 1 = placebo control group), and their interaction to predict chocolate consumption quantity and frequency

	Chocolate consumption quantity				Chocolate consumption frequency			
	B	*CI*	*SE*	*p*	*B*	*CI*	*SE*	*p*
**Fixed Parts**								
(Intercept)	24.9	19.7 – 30.1	2.66	<.001	21.6	16.8 – 26.4	2.44	<.001
Days	3.86	−0.64 – 8.36	2.30	.097	3.41	−0.99 – 7.81	2.25	.132
Group	2.18	−5.18 – 9.53	3.75	.564	1.30	−5.44 – 8.04	3.44	.706
Days × Group	−0.92	−7.35 – 5.52	3.28	.781	−0.74	−7.03 – 5.54	3.21	.817
**Random Parts**								
σ^2	382				365			
$\tau_{00, \text{participants}}$	158				123			
$N_{\text{participants}}$	70				70			
Observations	580				580			
R^2 / Ω_0^2	.374 / .356				.336 / .314			

Table S6

Standardized estimates (B), confidence intervals (CI), standard errors (SE), and p-values for the linear mixed models with the predictors days (0 = rest day, 1 = intervention day), group (0 = experimental group, 1 = placebo control group), pretest scores of the Food Cravings Questionnaire–Trait–reduced (chocolate version), and their interactions to predict chocolate consumption quantity and frequency

	Chocolate consumption quantity				Chocolate consumption frequency			
	B	*CI*	*SE*	*p*	*B*	*CI*	*SE*	*p*
**Fixed Parts**								
(Intercept)	25.7	21.4 – 30.0	2.18	<.001	22.1	18.0 – 26.2	2.10	<.001
Days	4.57	0.03 – 9.10	2.32	.051	4.30	−0.12 – 8.73	2.26	.059
Group	2.57	−3.43 – 8.57	3.06	.403	1.60	−4.17 – 7.37	2.94	.588
Food Cravings Questionnaire–Trait–reduced (chocolate version)	8.12	2.85 – 13.4	2.69	.003	5.51	0.45 – 10.6	2.58	.034
Days × Group	−1.60	−8.06 – 4.86	3.30	.628	−1.34	−7.64 – 4.96	3.21	.678
Days × Food Cravings Questionnaire–Trait–reduced (chocolate version)	5.10	−0.56 – 10.8	2.89	.080	6.45	0.92 – 12.0	2.82	.024
Group × Food Cravings Questionnaire–Trait–reduced (chocolate version)	2.39	−4.36 – 9.15	3.45	.489	1.87	−4.63 – 8.37	3.31	.573
Days × Group × Food Cravings Questionnaire–Trait–reduced (chocolate version)	−5.85	−13.1 – 1.44	3.72	.118	−4.18	−11.3 – 2.93	3.63	.251
**Random Parts**								
σ^2	381				362			
$\tau_{00,\ \text{participants}}$	74.6				66.7			
$N_{\text{participants}}$	70				70			
Observations	580				580			
R^2 / Ω_0^2	.359 / .349				.324 / .312			

Table S7

Standardized estimates (B), confidence intervals (CI), standard errors (SE), and p-values for the linear mixed models with the predictors days (0 = rest day, 1 = intervention day), group (0 = experimental group, 1 = placebo control group), pretest scores of the Restraint Scale, and their interactions to predict chocolate consumption quantity and frequency

	Chocolate consumption quantity				Chocolate consumption frequency			
	B	*CI*	*SE*	*p*	*B*	*CI*	*SE*	*p*
**Fixed Parts**								
(Intercept)	24.8	19.6 – 30.0	2.65	<.001	21.5	16.7 – 26.3	2.4	<.001
Days	3.77	−0.75 – 8.29	2.31	.106	3.36	−1.04 – 7.76	2.24	.138
Group	2.42	−4.90 – 9.73	3.73	.519	1.46	−5.26 – 8.18	3.43	.671
Restraint Scale	−1.99	−6.96 – 3.00	2.54	.435	−1.48	−6.06 – 3.10	2.34	.528
Days × Group	−0.70	−7.15 – 5.75	3.29	.832	−0.38	−6.66 – 5.90	3.20	.905
Days × Restraint Scale	−1.97	−6.31 – 2.36	2.21	.375	−1.36	−5.58 – 2.87	2.15	.531
Group × Restraint Scale	−0.05	−7.48 – 7.38	3.79	.990	1.45	−5.39 – 8.30	3.49	.678
Days × Group × Restraint Scale	0.18	−6.29 – 6.66	3.30	.956	−4.03	−10.3 – 2.27	3.21	.213
**Random Parts**								
σ^2	383				363			
$\tau_{00, \text{participants}}$	154				121			
$N_{\text{participants}}$	70				70			
Observations	580				580			
R^2 / Ω_0^2	.374 / .358				.341 / .322			

Table S8

Standardized estimates (B), confidence intervals (CI), standard errors (SE), and p-values for the linear mixed models with the predictors days (0 = rest day, 1 = intervention day), group (0 = experimental group, 1 = placebo control group), pretest scores of the Dutch Eating Behavior Questionnaire (restrained eating subscale), and their interactions to predict chocolate consumption quantity and frequency

	Chocolate consumption quantity				Chocolate consumption frequency			
	B	*CI*	*SE*	*p*	*B*	*CI*	*SE*	*p*
**Fixed Parts**								
(Intercept)	24.8	19.6 – 30.0	2.67	<.001	21.5	16.7 – 26.3	2.45	<.001
Days	3.87	−0.63 – 8.39	2.30	.096	3.43	−0.97 – 7.83	2.25	.130
Group	2.31	−5.07 – 9.69	3.77	.542	1.38	−5.38 – 8.14	3.45	.690
Dutch Eating Behavior Questionnaire (restrained eating subscale)	−2.26	−6.80 – 2.29	2.32	.334	−2.04	−6.21 – 2.12	2.13	.340
Days × Group	−0.99	−7.43 – 5.46	3.29	.765	−0.85	−7.14 – 5.44	3.21	.792
Days × Dutch Eating Behavior Questionnaire (restrained eating subscale)	−0.85	−4.67 – 2.97	1.96	.664	−0.41	−4.14 – 3.32	1.90	.829
Group × Dutch Eating Behavior Questionnaire (restrained eating subscale)	3.45	−4.11 – 11.0	3.86	.374	2.50	−4.42 – 9.43	3.54	.481
Days × Group × Dutch Eating Behavior Questionnaire (restrained eating subscale)	−1.04	−7.56 – 5.48	3.33	.755	−3.04	−9.41 – 3.32	3.25	.351
**Random Parts**								
σ^2	383				366			
$\tau_{00,\ participants}$	159				124			
$N_{participants}$	70				70			
Observations	580				580			
R^2 / Ω_0^2	.374 / .358				.338 / .317			

Figure caption

Figure S1. Means and standard errors of touching time displaying the three-way interaction *group* × *measurement* × *stimulus*. Participants reacted faster to pictures of food than to pictures of objects and reacted faster at posttest than at pretest, with larger decreases in touching times in the experimental and placebo control group than in the inactive control group. Following up the three-way interaction, however, was inconclusive: between-group comparisons showed that the experimental and placebo control group did not differ from each other in any touching times (all $t_{(68)}$s < 0.77, all ps > .446), did not differ from the inactive control group at pretest (all $t_{(68)}$s < 0.85, all ps > .400) but had faster touching times than the inactive control group at posttest for both food and objects (all $t_{(68)}$s > 2.59, all ps < .012); within-group comparisons showed that touching times decreased from pre- to posttest within each group and for both food and objects (all $t_{(34)}$s > 4.08, all ps < .001) and touching times were faster for food than for objects within each group and at both pretest and posttest (all $t_{(34)}$s > 3.51, all ps < .002).

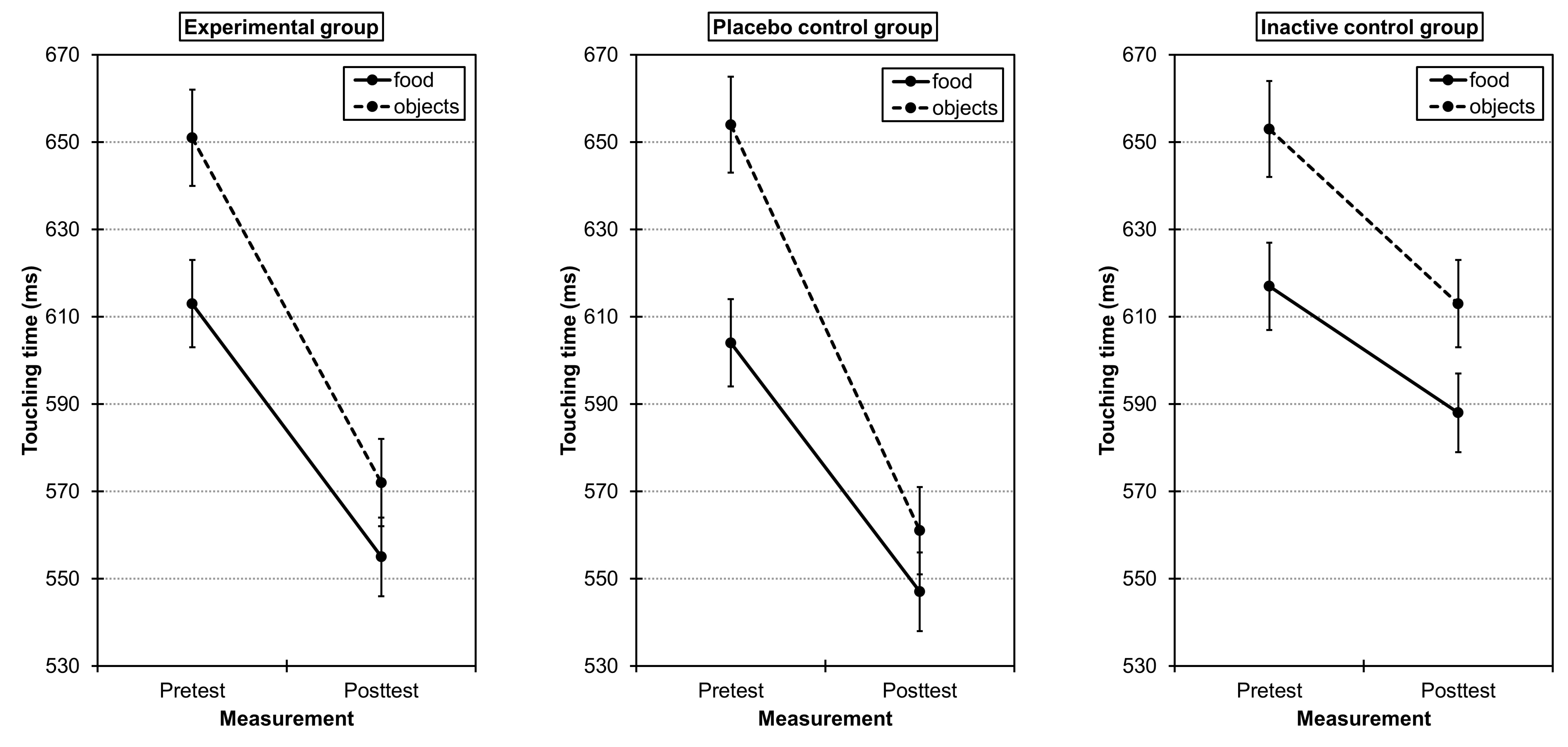

Experimental group
Placebo control group
Inactive control group
food
objects
Touching time (ms)
670
650
630
610
590
570
550
530
Pretest
Posttest
Measurement
