# Supplementary material for: Effects of a Smartphone-Based Approach-Avoidance Intervention on Chocolate Craving and Consumption: Randomized Controlled Trial
Source: JMIR Mhealth Uhealth. 2019 Nov 1;7(11):e12298. doi: 10.2196/12298 (PMC6858611; doi:10.2196/12298)
Supplement: Multimedia Appendix 6 [file mhealth_v7i11e12298_app6.pdf]

| CONSORT-EHEALTH Checklist V1.6.2 Report | Manuscript Number | 12298 |
|---|---|---|

**(based on CONSORT-EHEALTH V1.6), available at [http://tinyurl.com/consort-ehealth-v1-6].**

**Date completed**

10/29/2019 11:03:19

**by**

Adrian Meule

Effects of a Smartphone-Based Approach-Avoidance Intervention on Chocolate Craving and Consumption: Randomized Controlled Trial

## TITLE

**1a-i) Identify the mode of delivery in the title**

"smartphone"

**1a-ii) Non-web-based components or important co-interventions in title**

not applicable

**1a-iii) Primary condition or target group in the title**

"Chocolate Craving and Consumption"

## ABSTRACT

**1b-i) Key features/functionalities/components of the intervention and comparator in the METHODS section of the ABSTRACT**

"Within a 10-day period, regular chocolate eaters (n=105, 86% female) performed 5 sessions during which they continuously avoided (ie, swiped upward) chocolate stimuli (experimental group, n=35), performed 5 sessions during which they approached and avoided chocolate stimuli equally often (placebo control group, n=35), or did not perform any training sessions (inactive control group, n=35). Training effects were measured during laboratory sessions before and after the intervention period and further continuously through daily ecological momentary assessment."

**1b-ii) Level of human involvement in the METHODS section of the ABSTRACT**

"Within a 10-day period, regular chocolate eaters (n=105, 86% female) performed 5 sessions during which they continuously avoided (ie, swiped upward) chocolate stimuli (experimental group, n=35), performed 5 sessions during which they approached and avoided chocolate stimuli equally often (placebo control group, n=35), or did not perform any training sessions (inactive control group, n=35). Training effects were measured during laboratory sessions before and after the intervention period and further continuously through daily ecological momentary assessment."

**1b-iii) Open vs. closed, web-based (self-assessment) vs. face-to-face assessments in the METHODS section of the ABSTRACT**

"Within a 10-day period, regular chocolate eaters (n=105, 86% female) performed 5 sessions during which they continuously avoided (ie, swiped upward) chocolate stimuli (experimental group, n=35), performed 5 sessions during which they approached and avoided chocolate stimuli equally often (placebo control group, n=35), or did not perform any training sessions (inactive control group, n=35). Training effects were measured during laboratory sessions before and after the intervention period and further continuously through daily ecological momentary assessment."

**1b-iv) RESULTS section in abstract must contain use data**

"Self-reported chocolate craving and consumption as well as body fat mass significantly decreased from pre- to postmeasurement across all groups. Ecological momentary assessment reports evidenced no differences in chocolate craving and consumption between intervention days and rest days as a function of the group."

**1b-v) CONCLUSIONS/DISCUSSION in abstract for negative trials**

"A smartphone-based approach-avoidance training did not affect eating-related and anthropometric measures over and above measurement-based changes in this study. Future controlled studies need to examine whether other techniques of modifying food approach tendencies show an add-on benefit over conventional, monitoring-based intervention effects."

## INTRODUCTION

**2a-i) Problem and the type of system/solution**

I don't want to do this anymore. I am sitting at completing this stupid questionnaire for like an hour already. I find it unacceptable that JMIR is forcing me to do this after the manuscript has already been accepted for publication. I don't think that anybody will read this here anyway.

**2a-ii) Scientific background, rationale: What is known about the (type of) system**

-

**Does your paper address CONSORT subitem 2b?**

-

## METHODS

**3a) CONSORT: Description of trial design (such as parallel, factorial) including allocation ratio**

-

**3b) CONSORT: Important changes to methods after trial commencement (such as eligibility criteria), with reasons**

-

**3b-i) Bug fixes, Downtimes, Content Changes**

-

**4a) CONSORT: Eligibility criteria for participants**

-

**4a-i) Computer / Internet literacy**

-

**4a-ii) Open vs. closed, web-based vs. face-to-face assessments:**

-

**4a-iii) Information giving during recruitment**

-

**4b) CONSORT: Settings and locations where the data were collected**

-

**4b-i) Report if outcomes were (self-)assessed through online questionnaires**

-

**4b-ii) Report how institutional affiliations are displayed**

-

**5) CONSORT: Describe the interventions for each group with sufficient details to allow replication, including how and when they were actually administered**

**5-i) Mention names, credential, affiliations of the developers, sponsors, and owners**

-

**5-ii) Describe the history/development process**

-

**5-iii) Revisions and updating**

-

**5-iv) Quality assurance methods**

-

**5-v) Ensure replicability by publishing the source code, and/or providing screenshots/screen-capture video, and/or providing flowcharts of the algorithms used**

-

**5-vi) Digital preservation**

-

**5-vii) Access**

-

**5-viii) Mode of delivery, features/functionalities/components of the intervention and comparator, and the theoretical framework**

-

**5-ix) Describe use parameters**

-

**5-x) Clarify the level of human involvement**

-

**5-xi) Report any prompts/reminders used**

-

**5-xii) Describe any co-interventions (incl. training/support)**

-

**6a) CONSORT: Completely defined pre-specified primary and secondary outcome measures, including how and when they were assessed**

| | | |
|---|---|---|
| these information is explained at length in the manuscript | | |
| **6a-i) Online questionnaires: describe if they were validated for online use and apply CHERRIES items to describe how the questionnaires were designed/deployed** | | |
| | | |
| **6a-ii) Describe whether and how "use" (including intensity of use/dosage) was defined/measured/monitored** | | |
| | | |
| **6a-iii) Describe whether, how, and when qualitative feedback from participants was obtained** | | |
| | | |
| **6b) CONSORT: Any changes to trial outcomes after the trial commenced, with reasons** | | |
| - | | |
| **7a) CONSORT: How sample size was determined** | | |
| **7a-i) Describe whether and how expected attrition was taken into account when calculating the sample size** | | |
| we did not expect large attrition rates | | |
| **7b) CONSORT: When applicable, explanation of any interim analyses and stopping guidelines** | | |
| these information is explained at length in the manuscript | | |
| **8a) CONSORT: Method used to generate the random allocation sequence** | | |
| the experimenters did it with https://www.randomizer.org/ | | |
| **8b) CONSORT: Type of randomisation; details of any restriction (such as blocking and block size)** | | |
| no restrictions | | |
| **9) CONSORT: Mechanism used to implement the random allocation sequence (such as sequentially numbered containers), describing any steps taken to conceal the sequence until interventions were assigned** | | |
| the experimenters did it with https://www.randomizer.org/ | | |
| **10) CONSORT: Who generated the random allocation sequence, who enrolled participants, and who assigned participants to interventions** | | |
| the experimenters did it with https://www.randomizer.org/ | | |
| **11a) CONSORT: Blinding - If done, who was blinded after assignment to interventions (for example, participants, care providers, those assessing outcomes) and how** | | |
| **11a-i) Specify who was blinded, and who wasn't** | | |
| "The study was advertised as a study on "automatic reactions to chocolate-containing foods in daily life." That is, participants were not informed that the aim of the study was to change chocolate craving and consumption." | | |
| **11a-ii) Discuss e.g., whether participants knew which intervention was the "intervention of interest" and which one was the "comparator"** | | |
| "The study was advertised as a study on "automatic reactions to chocolate-containing foods in daily life." That is, participants were not informed that the aim of the study was to change chocolate craving and consumption." | | |
| **11b) CONSORT: If relevant, description of the similarity of interventions** | | |
| - | | |
| **12a) CONSORT: Statistical methods used to compare groups for primary and secondary outcomes** | | |
| all analyses are explained at length in the manuscript | | |
| **12a-i) Imputation techniques to deal with attrition / missing values** | | |
| there were almost no missing values, it is explicitly stated in the figure caption of figure 1 that sample size was slightly smaller for two variables, that is, missing values were not imputed | | |
| **12b) CONSORT: Methods for additional analyses, such as subgroup analyses and adjusted analyses** | | |
| all analyses are explained at length in the manuscript | | |
| RESULTS | | |
| **13a) CONSORT: For each group, the numbers of participants who were randomly assigned, received intended treatment, and were analysed for the primary outcome** | | |
| figure 1 | | |
| **13b) CONSORT: For each group, losses and exclusions after randomisation, together with reasons** | | |
| figure 1 | | |
| **13b-i) Attrition diagram** | | |
| participant flow can be seen in figure 1 and compliance rates can be found in table 1 | | |
| **14a) CONSORT: Dates defining the periods of recruitment and follow-up** | | |
| readers can tell from the preregistration that the study was conducted in 2018; several times in the manuscript, it is stated that the intervention period between pre and posttest was 10 days | | |
| **14a-i) Indicate if critical "secular events" fell into the study period** | | |
| not applicable | | |
| **14b) CONSORT: Why the trial ended or was stopped (early)** | | |
| not applicable, the sample size was collected in line with the power analysis | | |
| **15) CONSORT: A table showing baseline demographic and clinical characteristics for each group** | | |
| table 1 | | |
| **15-i) Report demographics associated with digital divide issues** | | |
| "The final sample comprised 105 participants (85.7% female, 90/105) with a mean age of 23.4 years (SD 5.07) and a mean body mass index of 23.3 kg/m² (SD 4.14). The majority of participants had German (52.4%, 55/105) or Austrian (40.0%, 42/105) citizenship and were university students (94.3%, 99/105)." | | |
| **16a) CONSORT: For each group, number of participants (denominator) included in each analysis and whether the analysis was by original assigned groups** | | |
| **16-i) Report multiple "denominators" and provide definitions** | | |
| everything of these things is explained in figure 1 and in the figure caption | | |
| **16-ii) Primary analysis should be intent-to-treat** | | |
| not applicable, there was only one person who discontinued participation | | |
| **17a) CONSORT: For each primary and secondary outcome, results for each group, and the estimated effect size and its precision (such as 95% confidence interval)** | | |
| effect sizes are reported throughout | | |
| **17a-i) Presentation of process outcomes such as metrics of use and intensity of use** | | |
| reported in table 1 | | |
| **17b) CONSORT: For binary outcomes, presentation of both absolute and relative effect sizes is recommended** | | |
| not applicable | | |
| **18) CONSORT: Results of any other analyses performed, including subgroup analyses and adjusted analyses, distinguishing pre-specified from exploratory** | | |
| "Analyses of variance for repeated measures with group (experimental vs placebo control vs inactive control) as between-subjects factor and measurement (pre- vs posttest) as within-subjects factor were calculated to examine changes in body mass index and body fat mass as a function of group. Moderation analyses were calculated with PROCESS [31] to examine whether FCQ-T-r scores at pretest, Restraint Scale scores, and DEBQ scores moderated any effects of group on chocolate consumption, body mass index, and body fat mass at posttest while controlling for pretest values. Restraint Scale scores and DEBQ scores were also tested as moderators of effects of group on FCQ-T-r scores at posttest while controlling for FCQ-T-r scores at pretest. Fisher exact tests were calculated to compare groups regarding the 2 debriefing questions. These analyses were not included in the preregistration protocol." | | |
| **18-i) Subgroup analysis of comparing only users** | | |
| not applicable, we did not do that, compliance was high | | |
| **19) CONSORT: All important harms or unintended effects in each group** | | |
| displayed in the participant flow chart in figure 1 | | |
| **19-i) Include privacy breaches, technical problems** | | |
| displayed in the participant flow chart in figure 1 | | |
| **19-ii) Include qualitative feedback from participants or observations from staff/researchers** | | |
| "A total of 93 participants (88.6%, 93/105) indicated that they thought the aim of the study was to assess their behavior in relation to chocolate, 4 participants (3.8%, 4/105) did not think so, and 8 participants (7.6%, 8/105) indicated that they did not know. There were no significant differences between groups ($\chi2=4.6$; $P=.30$; □=.224). A total of 29 participants (27.6%, 29/105) indicated that they thought the aim of the study was to change their behavior in relation to chocolate, 61 participants (58.1%, 61/105) did not think so, and 15 participants (14.3%, 15/105) indicated that they did not know. Here, responses did significantly differ between groups ($\chi2=9.63$; $P=.04$;□=.317): more participants in the inactive control group (n=26) did not think that the study's aim was to change their behavior than participants in both the experimental group (n=18) and the placebo control group (n=17), whereas the latter 2 groups did not differ from each other (based on follow-up z tests using alpha=.05)." | | |
| DISCUSSION | | |
| **20) CONSORT: Trial limitations, addressing sources of potential bias, imprecision, multiplicity of analyses** | | |
| **20-i) Typical limitations in ehealth trials** | | |

| | | |
|---|---|---|
| "Several other methodological considerations might account for these results. For example, although we selected food stimuli with which we have previously detected an approach bias in a comparable sample using a joystick-based task [23], it may be that approach-avoidance training work better when using personalized stimuli, that is, pictures of foods that participants actually crave and consume regularly in their daily life. In related research on attentional bias, for example, it has been found that internal reliability of reaction time tasks can be increased when personalized stimuli are used [42]. Furthermore, we used relatively few training sessions (5), which may have been insufficient to produce meaningful changes in approach bias and eating behavior. However, evidence from joystick-based approach-avoidance training suggest that few sessions suffice to detect such effects in relation to alcohol [43]. However, other smartphone-based studies did indeed use more frequent training sessions [12,13]. Thus, the number of training sessions required in smartphone-based approach-avoidance training need further examination. Finally, although we instructed participants regarding the meaning of upward and downward swipe movements, we did not assess whether they actually perceived the movements as pushing or pulling the pictures away from or toward themselves. Therefore, we cannot rule out the possibility that participants did not perceive the movements as intended, which could explain the lack of finding an approach bias and training effects." | | |
| **21) CONSORT: Generalisability (external validity, applicability) of the trial findings** | | |
| **21-i) Generalizability to other populations** | | |
| "Interpretation of results needs to consider the sample investigated in this study. Although we included both men and women with a body mass index ranging from underweight to obese, the majority of the sample were normal-weight women. It has been previously suggested that successful retraining of appetitive reactions and consumption behaviors may primarily be found in clinical samples." | | |
| **21-ii) Discuss if there were elements in the RCT that would be different in a routine application setting** | | |
| "Interpretation of results needs to consider the sample investigated in this study. Although we included both men and women with a body mass index ranging from underweight to obese, the majority of the sample were normal-weight women. It has been previously suggested that successful retraining of appetitive reactions and consumption behaviors may primarily be found in clinical samples." | | |
| **22) CONSORT: Interpretation consistent with results, balancing benefits and harms, and considering other relevant evidence** | | |
| **22-i) Restate study questions and summarize the answers suggested by the data, starting with primary outcomes and process outcomes (use)** | | |
| "This study examined effects of a smartphone-based approach-avoidance intervention on approach bias toward chocolate-containing foods and chocolate craving/consumption relative to placebo and no training conditions. The 3 groups were well matched at baseline, treatment adherence was high (87% completed training sessions), and study attrition was low. All dependent measures evidenced good-to-excellent reliability. However, a smartphone-based AAT did neither reveal an approach bias toward chocolate-containing foods at baseline nor a modulation through training. In fact, chocolate craving and consumption decreased throughout the study period in all 3 groups. This self-report finding was corroborated in that participants in all groups lost body fat. Crucially, only a minority of participants thought that this study's aim was to change their behavior, suggesting that these effects were not because of demand characteristics. Comparing chocolate craving and consumption on intervention versus rest days did not reveal any short-term effects of the training." | | |
| **22-ii) Highlight unanswered new questions, suggest future research** | | |
| "future research needs to determine whether other techniques such as moving the smartphone toward and away with arm movements [36,37] or using tilt movements [38] are better suited for detecting and changing approach-avoidance tendencies with smartphones. In addition, it has recently been found that combining approach-avoidance actions with affective feedback produced stronger changes in food choices than conventional approach-avoidance training [39]. Thus, using such consequence-based approach-avoidance training may similarly enhance training effects with smartphone-based implementations." | | |
| Other information | | |
| **23) CONSORT: Registration number and name of trial registry** | | |
| https://aspredicted.org/pt9df.pdf | | |
| **24) CONSORT: Where the full trial protocol can be accessed, if available** | | |
| multimedia appendix 1 | | |
| **25) CONSORT: Sources of funding and other support (such as supply of drugs), role of funders** | | |
| funded by the European Research Council, which had no influence on study design, data collection, data analyses, and interpretation of results | | |
| **X26-i) Comment on ethics committee approval** | | |
| "The study was approved by the ethical review board of the University of Salzburg" | | |
| **x26-ii) Outline informed consent procedures** | | |
| "At pretest, participants signed informed consent" | | |
| **X26-iii) Safety and security procedures** | | |
| not applicable, this was not a clinical sample | | |
| **X27-i) State the relation of the study team towards the system being evaluated** | | |
| no conflict of interest | | |
